# Anti-Idiotypic mRNA Vaccine to Treat Autoimmune Disorders

**DOI:** 10.3390/vaccines12010009

**Published:** 2023-12-21

**Authors:** Sarfaraz K. Niazi

**Affiliations:** College of Pharmacy, University of Illinois, Chicago, IL 60012, USA; niazi@niazi.com; Tel.: +1-312-297-0000

**Keywords:** mRNA, vaccines, autoantibodies, autoantigens, anti-idiotypic antibodies, autoimmune disorders (ADs), diabetes type 1

## Abstract

The 80+ existing autoimmune disorders (ADs) affect billions with little prevention or treatment options, except for temporary symptomatic management, leading to enormous human suffering and a monumental financial burden. The autoantibodies formed in most ADs have been identified, allowing the development of novel anti-idiotypic antibodies to mute the autoantibodies using vaccines. Nucleoside vaccines have been successfully tested as antigen-specific immunotherapies (ASI), with mRNA technology offering multi-epitope targeting to mute multiple autoantibodies. This paper proposes using mRNA technology to produce anti-idiotypic antibodies with broad effectiveness in preventing and treating them. This paper delves into the state-of-the-art mRNA design strategies used to develop novel ASIs by selecting appropriate T cell and B cell epitopes to generate anti-idiotypic antibodies. The low cost and fast development of mRNA vaccines make this technology the most affordable for the global control of ADs.

## 1. Introduction

The term “vaccine” means any substance designed to be administered to a human being for the prevention of one or more diseases, according to 26 USC § 4132(a)(2); thus, a product that creates an immune response to alleviate an autoimmune disorder can be labeled as a vaccine. Vaccines are diverse in their approaches to stimulating immune responses against specific pathogens. Inactivated or killed vaccines, like the polio vaccine, utilize pathogens that have been rendered noninfectious [1]. Live attenuated vaccines, exemplified by the MMR vaccine, contain weakened forms of the pathogen [1]. Subunit, recombinant, or protein vaccines, such as the hepatitis B vaccine, include only a portion of the pathogen’s antigen [2]. Viral vector vaccines, like those developed by Johnson & Johnson and AstraZeneca for COVID-19, employ harmless viruses to deliver genetic material from the target pathogen [3,4].

DNA vaccines, represented by research on HIV, introduce circular DNA encoding part of the pathogen’s antigen [5]. Lastly, mRNA vaccines, including the Pfizer-BioNTech and Moderna COVID-19 vaccines, instruct cells to produce pathogen proteins via messenger RNA [6], showcasing the diverse strategies employed in modern vaccine development. Moderna is developing mRNA in combination with Keytruda as a new treatment for cancer [7].

**Figure 1:**
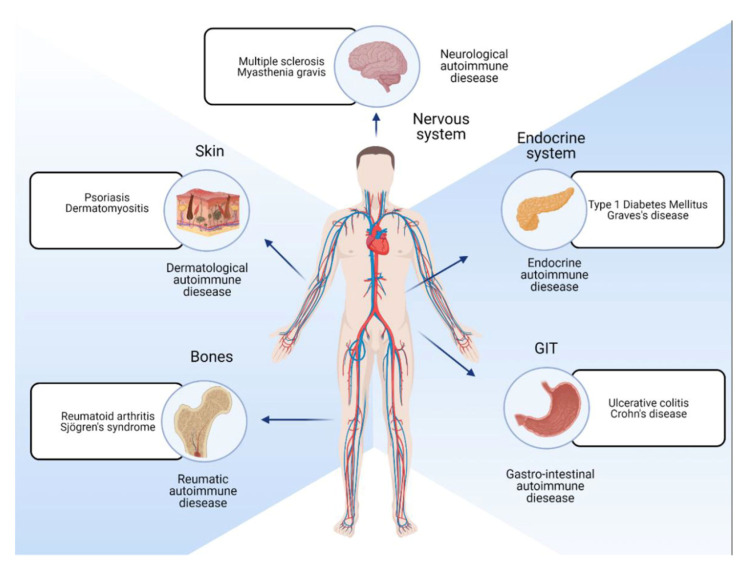
Autoimmunity on Wikipedia. https://en.wikipedia.org/wiki/Autoimmunity (accessed on 13 November 2023).

While preventing infections has been the mainstream of vaccines, the focus is now turning toward autoimmune disorders (ADs) that occur when the immune system mistakenly attacks the body’s tissues, leading to various chronic and debilitating conditions (ADs) [8] that bring enormous suffering to humanity (Figure 1). This was one message given by Drew Weissman [9] in the 2023 Physiology and Medicine lecture about how we can now effectively treat all autoimmune disorders.

The binding of autoantibodies to autoantigens, a hallmark of autoimmune diseases, involves the immune system mistakenly identifying its proteins, DNA, or RNA as foreign. This misrecognition triggers the production of autoantibodies, which are structurally and functionally similar to normal antibodies but target the body’s tissues [10]. These autoantibodies bind to autoantigens through molecular complementarity, where their variable regions (paratopes) specifically adhere to epitopes on the autoantigens. This interaction, dictated by the shape, charge, and hydrophobic or hydrophilic properties, can induce conformational changes in autoantibodies and autoantigens, potentially disrupting normal cellular functions [11]. The subsequent immune response, including complement pathway activation and inflammatory cytokine release, leads to the tissue damage characteristics of autoimmune conditions like rheumatoid arthritis [12]. The exact mechanisms triggering autoantibody production are not entirely clear, but a combination of genetic, environmental, and other factors is believed to contribute to the onset of autoimmunity [13,14].

Autoantibodies are predictable biomarkers of ADs such as type 1 diabetes (anti-Gad antibodies), rheumatoid arthritis (RA) (HLA-DR4 allele, anti-citrullinated protein antibodies (ACPAs)), multiple sclerosis (MS) (genetic markers, vitamin D deficiency, MRI scans), celiac disease (HLA-DQ2 or HLA-DQ8 alleles), systemic lupus erythematosus (SLE) (antinuclear antibodies (ANAs)), and Graves’ disease and Hashimoto’s thyroiditis (thyroid peroxidase antibodies (TPOAb) and thyroglobulin antibodies (TgAb)). These autoantibodies can be monoclonal or polyclonal, with much diversity among the patients [15], and they may interact with multiple receptors; multiple autoantibodies can be responsible for one autoimmune disorder. Currently, 83 studies are reported on clinicaltrials.gov for autoimmune interventional trials using mRNA expression.

The world’s second best-selling drug in 2022 was AbbVie’s Humira. It is designed to treat rheumatoid arthritis, a progressive disease that causes stiffness, swelling, joint pain, and symptoms like a lack of energy. Psoriasis is another severe autoimmune disease in which scaly skin patches appear on the scalp, elbows, and knees. It can cover a large proportion of the body in severe cases. Other autoimmune severe diseases include multiple sclerosis (MS), type-1 diabetes, inflammatory bowel diseases (IBDs, which include Crohn’s disease and ulcerative colitis), and lupus (which causes joint pain and rashes on skin exposed to the sun). MS is a potentially disabling disease caused by the immune system attacking the outer sheath of nerve fibers and interrupting communication between the brain and other body parts. In advanced cases, patients lose the ability to walk. There are also rarer autoimmune diseases, such as myasthenia gravis, which cause weakness in the eye, face, and mouth muscles, causing blurred vision and difficulties chewing and speaking. 

There are over 80 autoimmune diseases, with symptoms ranging from relatively minor to disabling. About 10% of people globally suffer from autoimmune diseases: 13% of women and 7% of men. In 2022, almost 25% of the top 50 best-selling drugs worldwide were for various autoimmune diseases, with sales of more than USD 2.3 bn per year. The global market for drugs to treat autoimmune diseases will grow to USD 185 bn by 2029, with the US accounting for half the total. These costs also bring the issue of affordability of these drugs that highlights the need to develop alternate treatments that are supported by major global organizations such as the World Health Organization (WHO), United Nations (UN), UNICEF, UNAIDS, UNHCR, the World Bank, UNESCO, and many other charity organizations [16]. 

Vaccines specifically tested against autoimmune disorders remain an ongoing area of research and development. While vaccines have proven highly effective in preventing infectious diseases, their application in treating or preventing autoimmune disorders is a complex challenge. Experimental autoimmune disease models and clinical trials have explored the concept of therapeutic vaccines designed to modulate immune responses in autoimmune conditions like multiple sclerosis (MS). 

While, so far, no vaccine against ADs has been licensed, researchers are investigating novel approaches, including antigen-specific immunotherapies (ASIs) and tolerance-inducing vaccines, to redirect the immune system away from attacking the body’s tissues [17,18], and going through the maze of the etiology of ADs such genetic or environmental influences [19,20], immunological factors [21], hormonal influences that lead to a much higher incidence in women [22], and gut microbiome influence [23]. 

These strategies to manage ADs include B cell depletion therapy [24], where the immunoglobulin (Ig) light chains are replaced in these antibodies, primarily with Igλ and Igκ chains. Most naturally occurring autoantibodies can be effectively silenced by light chain exchange, with Igλ chains showing greater efficacy than Igκ chains as silencers [25].

Plasma exchange (plasmapheresis) removes autoantibodies from the blood. It is used in various autoimmune disorders, such as myasthenia gravis, and temporarily relieves symptoms [26]. Medications like corticosteroids and cyclophosphamide can suppress the overall immune response, thereby reducing the production of autoantibodies. However, these drugs do not specifically target autoantibodies and can have significant side effects [27]. These modalities are effective, but they require careful monitoring for side effects and are often adjusted based on individual patient responses. The choice of therapy depends on the specific autoimmune disease, its severity, the patient’s overall health, and other factors [28].

Drugs like Belimumab, which inhibit the B-lymphocyte stimulator (BLyS) protein, can reduce the activity of B cells and lower the production of autoantibodies in diseases like systemic lupus erythematosus [29]. For instance, mAbs such as rituximab target CD20-positive B cells, offering a promising approach in conditions like rheumatoid arthritis and multiple sclerosis [30]. Another class of medications, biologic agents like etanercept, infliximab, and adalimumab, specifically targets tumor necrosis factor (TNF), a key player in inflammation. These drugs have revolutionized the treatment of autoimmune conditions like rheumatoid arthritis but come with a risk of increased infections and certain skin cancers. Abatacept works by blocking the activation of T cells, a type of white blood cell involved in autoimmune responses.

Another area of interest is the modulation of immune checkpoints, crucial in maintaining immune tolerance. Drugs that manipulate these checkpoints have shown potential in treating ADs like type 1 diabetes [31]. Additionally, small molecule inhibitors, which can precisely target specific pathways in the immune response, are being investigated. JAK inhibitors, for example, have been effective in treating rheumatoid arthritis by inhibiting the Janus kinase pathway involved in the inflammatory process [32]. 

Cell-based therapies, including regulatory T-cell therapy, are also being explored to restore immune tolerance in ADs [33].

Personalized medicine, particularly in the context of neoantigen-based vaccines, is being researched. These vaccines are tailored to an individual’s specific autoimmune pathology, aiming to induce tolerance to autoantigens [34]. Gut microbiome modulation, as a therapeutic strategy, highlights the interconnectedness of the immune system and other bodily systems [35].

Recently, the idea of reducing immunogenicity by administering the epitopes of the neoantigen protein along with sugars to prompt the liver to identify it as a natural protein, and consequently suppress the abnormal antibodies, has been under development [36]. Inducing antigen-specific tolerance during an established immune response typically requires non-specific immunosuppressive signaling molecules such as a polymer glycosylated with N-acetylgalactosamine (pGal) and conjugated to the antigen via a self-immolating linker that allows for the dissociation of the antigen on endocytosis and its presentation in the immunoregulatory environment. 

## 2. Anti-Idiotypic Antibodies

The concept of antibodies against autoantibodies, anti-idiotypic antibodies, has garnered significant interest in the field of immunology and autoimmune disease treatment. These antibodies are designed to counteract the harmful effects of autoantibodies, which mistakenly target the body’s tissues. 

Anti-idiotypic antibodies are a unique class of antibodies that target another antibody’s idiotype, the specific antigen-binding part. These antibodies are integral to the immune system’s regulation, as they bind to the variable regions of other antibodies, particularly those typically involved in antigen binding [37]. The concept of anti-idiotypic antibodies is deeply rooted in the idiotypic network theory, which posits that antibodies can generate a response that produces these specialized antibodies against their idiotype [38]. Their potential therapeutic application, especially in autoimmune diseases, lies in their ability to neutralize and regulate autoantibodies, antibodies that mistakenly target the body’s tissues. By targeting these autoantibodies, anti-idiotypic antibodies can offer a novel form of immunotherapy with the possibility of minimal side effects and prolonged immunity [39]. Anti-idiotypic antibodies can regulate neutralizing autoantibodies and inhibit their secretion. This dual action underscores their potential utility in managing autoimmunity [40]. These antibodies can recognize antibody molecules with similar or identical structures within their variable regions, typically involved in antigen binding. This specificity is critical to their effectiveness in targeting problematic autoantibodies [37].

## 3. Nucleoside Vaccines

Plasmid DNA [41] and mRNA [5] vaccines have garnered high interest as a multi-utility platform suitable for prevention and therapy, from personalized medicine to global health solutions. Both can be quickly developed and manufactured using non-proprietary technologies. The product design comes directly from the genetic sequence of the desired protein, whether the origin of the protein is human or from a pathogen, opening the door to prevent and treat autoimmune disorders and infections. 

While mRNA vaccines have been licensed for human use, DNA vaccines are still awaiting first approval. Initially, there was much less interest in mRNA compared to plasmid DNA due to the transient nature of mRNA because of its instability; for example, only one out of 10,000 molecules of mRNA will escape an endosome into the cytoplasm [42]. However, this can also be an asset for the process whereby organisms control the production of desired proteins, which is due to RNAses that are widely present [43]. RNA has long been known to be an immunologically active molecule. Plasmid DNA, with its gene therapy applications, and the provision of a missing or defective protein with no immune responses against either the protein or the vector delivery system, is the main reason for using plasmid DNA.

### 3.1. DNA Vaccine

DNA vaccines use a small, circular piece of DNA, typically a plasmid, to stimulate an immune response against specific pathogens, triggering both humoral and cellular immunity that can be useful in cancer therapies. DNA vaccines introduce genetic material encoding a portion of the target pathogen’s antigen into host cells, where the cells produce the antigen, leading to an immune response. DNA vaccines have shown promise in preclinical and clinical studies for diseases like HIV, influenza, and COVID-19 [5,44,45]. 

Both DNA vectors (which are based on bacterial plasmids) and in vitro transcribed mRNA activate the innate immune system. DNA plasmids do so via their CpG motifs, which stimulate TLR9 [46]. The double-stranded structure of the DNA plasmid is also an immune stimulant through non-TLR mechanisms. It also acts on the TBK1-STING pathway through cytosolic receptors [47]. This results in the generation of Type 1 interferons, which act as adjuvants for generating immune responses against the antigen(s) encoded by the plasmid DNA vaccine.

The incorporation of a cDNA coding for the pro-apoptotic protein BAX into plasmid DNA coding for a secreted form of the pancreatic beta-cell antigen glutamic acid decarboxylase (GAD) was shown to promote the prevention of type 1 diabetes in non-obese diabetic (NOD) mice [41]. Since T1D starts with autoimmunity to one of these islet autoantigens, there is likely to be epitope spreading, and it may require a multipronged approach to ‘reverse’ autoimmunity to multiple antigens [48] that can be controlled by using multiple plasmids. Thus, it might be possible to pulse an individual with either anti-CD3 or anti-CD20 therapy and then start a tolerizing immune regimen with an approach like the DNA proinsulin plasmid.

The utility of the technology underlying the construction of the DNA-tolerizing plasmid is that it is relatively easy to modify its backbone and interchange different coding regions [49]. Thus, it is possible to arm the plasmid with constructs for suppressive cytokines like IL-4 and IL-10 or to co-administer plasmids encoding these cytokines. The number of immune suppressive GpG hexanucleotides can be expanded or even given as a separate oligonucleotide construct. Plasmids encoding other islet antigens can be provided separately, or constructs can be engineered in tandem. The DNA tolerizing plasmids are operative in animal models when given in conjunction with anti-CD20 therapy, thus allowing a two-pronged “knockout” punch with anti-CD20 to clear the deck, followed by antigen-specific tolerization. Overall, further clinical trials with tolerizing DNA plasmids to proinsulin and other islet cell autoantigens remain a worthy pursuit to potentially treat, prevent, and perhaps even ‘cure’ or ‘prevent’ T1D [46].

Vaccine technology to suppress, rather than prime, antigen-specific immune responses [42] has been validated in mouse models of multiple sclerosis, where a modified mRNA encoding a self-antigen delivered in a non-inflammatory lipoplex carrier led to the dampening of autoimmunity through the activation of antigen-specific regulatory T cells. Although further research is needed to assess the strategy’s clinical potential, a successful tolerizing vaccination approach could transform the treatment of autoimmune diseases, allergies, and allogeneic transplantation.

Clinical trials have further confirmed the role of nucleoside vaccines. For example, proinsulin is a major target of adaptive immunity in T1D. Engineered DNA plasmid encoding proinsulin (BHT-3021) preserves *β* cell function in T1D patients through a reduction in insulin-specific T cells when comparing the islet-specific CD8+ T cell frequencies assessed with multimers of monomeric human leukocyte antigen class I molecules loaded with peptides containing pancreatic or unrelated antigens. No serious adverse events related to BHT-3021 occurred. Proinsulin-reactive CD8+ T cells, but not T cells against unrelated islet or foreign molecules, decline in the BHT-3021 arm, demonstrating that a plasmid encoding proinsulin reduces the frequency of CD8+ T cells reactive to proinsulin while preserving C-peptide throughout dosing [50].

The clinical efficacy of plasmid-DNA products in muting autoantibodies has validated the role of nucleoside vaccines [51], expediting the development of mRNA vaccines now that they have been licensed, and ensuring their safety and efficacy.

The immune system naturally has mechanisms to prevent autoimmunity, known as immune tolerance. This system can sometimes fail, leading to ADs. Therapies designed to induce or restore this tolerance to self-antigens are being studied as potential treatments. One approach involves using regulatory T cells (Tregs) that have been expanded or modified outside the body and then reintroduced to suppress autoimmunity. Another approach is using drugs or agents that promote the generation or function of Tregs inside the body [52]. Another approach might involve using mRNA to express antigens to promote immune tolerance rather than an immune response, potentially reducing the production of harmful autoantibodies. Some proteins naturally suppress immune responses. mRNA can be designed to produce such immune suppressors, which, when expressed, might calm an overactive immune response or reduce excessive antibody production in ADs. 

### 3.2. mRNA Vaccines

mRNA vaccines offer a significant advantage in combating infectious diseases, mainly due to their ability to incorporate multiple epitopes, creating a broad and effective immune response. mRNA vaccines can carry multiple epitopes since they do not cluster or bind together to broaden the targets [53]. Multi-epitopes can include epitopes recognized by multiple MHC Class I and Class II molecules, potentially activating a broader range of immune responses and providing more comprehensive protection against a pathogen [54,55,56]. Reduced risk is also a chemical product reducing allergic risk [57]. 

Base modifications such as replacing uridine with pseudouridine in the mRNA significantly reduce the inflammatory response [58,59], a finding that resulted in the 2023 Nobel Prize being awarded to Katalin Karikó and Drew Weissman. Upon entering cells, unmodified IVT mRNA becomes intrinsically immunogenic with the activation of toll-like receptors (TLRs) that can recognize double-stranded viral RNA, the mechanisms behind this induction of the immune response. However, replacement with Ψ also requires the co-administration of an adjuvant such as lipopolysaccharide or an immunostimulatory oligo. In this regard, lipid nanoparticles play an immunoadjuvant role as carriers and adjuvants. The Ψ-modified mRNA is also more resistant to RNase L-mediated degradation [60]. A classic example of ignoring scientific facts came from a total failure of the CureVac COVID-19 mRNA vaccine, where no modifications were made, considering this unnecessary [61]. However, these modifications are irrelevant when the translated protein is innate and not antigenic [62].

The number of protein molecules translated from one mRNA molecule varies based on the length of the mRNA, translation efficiency, and the stability of the resulting protein. Also, multiple ribosomes can simultaneously translate the same mRNA molecule, forming a chain of ribosomes known as polysomes [63]. The number of ribosomes that can translate mRNA simultaneously is determined by factors such as ribosome availability, cellular conditions, and specific mRNAs and their associated regulatory elements [63]. 

Compared to other vaccine platforms like live attenuated and subunit vaccines, mRNA vaccines have been noted for their safety profile, efficacy, and ease of production. Their synthetic nature allows for rapid development and scalability, which is particularly crucial in responding to emerging infectious diseases [53].

During the 1980s, efficient methods for producing mRNA without cell culture, called in vitro transcription, were introduced. This decisive step accelerated the development of molecular biology applications in several fields. Ideas of using messenger RNA (mRNA) technologies for vaccine and therapeutic purposes also took off, but roadblocks lay ahead. In vitro transcribed mRNA was considered unstable and challenging to deliver, requiring the development of sophisticated carrier lipid systems to encapsulate the mRNA.

The idea of using mRNA technology to target autoantibodies might seem counterintuitive at first, as the primary therapeutic application of mRNA technology to date has been the development of mRNA vaccines that induce the production of antibodies against specific antigens.

However, mRNA technology can be applied innovatively, including the possible targeting or modulation of antibodies or immune responses. The immune system has intrinsic mechanisms for regulating antibody production, including feedback inhibition. For example, when high-affinity antibodies bind to their specific antigens, they can provide negative feedback to B cells, preventing further activation. mRNA could be leveraged to augment these natural regulatory mechanisms by encoding molecules or factors that enhance feedback inhibition.

Apoptosis, or programmed cell death, is a natural mechanism to regulate cell populations. mRNA could be engineered to express pro-apoptotic molecules specifically in targeted B cells or plasma cells, leading to their selective elimination and thus reducing antibody production. 

B cells can be activated through their B cell receptor (BCR) and toll-like receptors (TLRs). mRNA could be harnessed to express molecules that interfere with these receptors or their signaling pathways, modulating the B cell response [64]. Immune checkpoints regulate the amplitude of immune responses. Some of these checkpoints play roles in B cell activation and function. mRNA could overexpress these checkpoint molecules or their ligands to dampen B cell activation and subsequent antibody production [65].

mRNA to Modulate Immune Cell Behavior: mRNA could be potentially used to express molecules that alter the behavior of immune cells, such as B cells (which produce antibodies) or regulatory T cells, to modulate immune responses [66].

mRNA could produce molecules that interfere with or block the action of specific antibodies. For instance, mRNA could code for proteins or peptides that bind to and neutralize autoantibodies in autoimmune conditions. By producing specific proteins or inhibitors, mRNA technology might modulate cytokine responses or intracellular signaling pathways in B cells that affect their activation, proliferation, and antibody production [5]. 

There is also a potential to leverage mRNA to modulate the behavior of regulatory T cells (Tregs). Tregs play a role in suppressing immune responses, and enhancing their function could indirectly modulate excessive antibody production in autoimmune conditions [67].

Plasma cells are B cells’ final differentiated form that produces antibodies. mRNA could potentially be used to express molecules that interfere with the survival or function of plasma cells, thereby reducing antibody production. This would be particularly valuable for diseases characterized by harmful autoantibody production. 

### 3.3. mRNA Design

**Figure 2:**
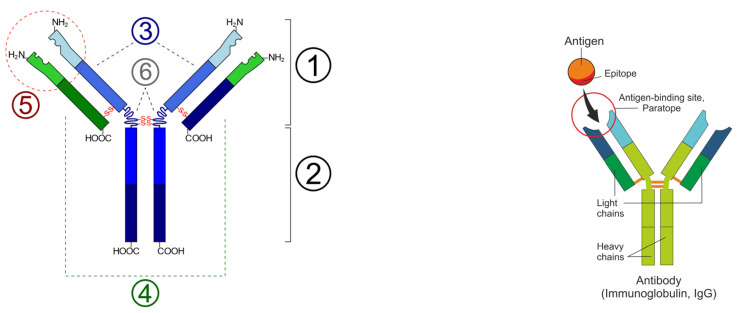
(**Left**): Schematic diagram of the basic unit of immunoglobulin (antibody); 1: Fab; 2: Fc; 3: heavy chain (consist of VH, CH1, hinge, CH2 and CH3 regions: from N-term); 4: light chain (consist of VL and CL regions: from N-term); 5: antigen binding site; 6: hinge regions; -S-S- mean disulfide bonds. [Wikimedia Commons contributors, “File: Immunoglobulin basic unit.svg,” Wikimedia Commons, https://commons.wikimedia.org/w/index.php?title=File:Immunoglobulin_basic_unit.svg&oldid=622136039 (accessed on 6 December 2023)] (**Right**): Epitope-paratope association (Shutterstock).

The first step in creating an mRNA vaccine is to identify the epitopes of the autoantigen concerning its specific autoantibody, and then to identify the paratopes of the autoantibody to determine the sequence of the antigen expressed by mRNA to produce an antibody against the autoantibody (Figure 2). A paratope, also known as an antigen-binding site, is the part of an antibody that recognizes and binds to an antigen. It is a small region at the tip of the antibody’s antigen-binding fragment, containing parts of its heavy and light chains. Each paratope comprises six complementarity-determining regions—three from each of the light and heavy chains—that extend from a fold of anti-parallel beta sheets. Each arm of the Y-shaped antibody has an identical paratope at the end.

The determination of paratopes in autoantibodies typically involves structural and computational analysis. There is not a standardized listing of paratopes for all autoantibodies because each autoantibody–antigen interaction is unique, and the specific paratope can vary from one autoantibody to another. The identity of reactants for autoantibodies has been successively refined from whole cellular organelles (immunofluorescence), identified molecules (immunoblot; gene expression libraries), and epitope regions (truncated cDNAs; peptide scanning) to contact residues, as described here. Most autoantibodies react with conformational epitopes, in which amino acids distant in the linear sequence come into contiguity via protein folding. Identifying contact sites with the antibody paratope requires technologies, crystallography, or the antibody screening of phage-displayed random peptide libraries. A more precise definition of the structure of conformational autoepitopes clarifies epitope mimicry, epitope spreading, molecular spatial relationships between B and T cell autoepitopes, and their impacts on novel diagnostic and therapeutic (vaccine) molecules [68].

The complex process of matching a sequence of the Fc region of an antibody to an epitope sequence involves the synergy between bioinformatics and molecular biology techniques. Discerning the distinct roles and structures of the Fc region and epitope is pivotal. The Fc region [69] interacts with cell surface receptors and complement proteins at an epitope, the specific portion of an antigen recognized by the immune system [70].

Each of these methods has its limitations, and, often, a combination of several approaches is used to accurately predict or infer paratope structures, the vast diversity of antibody structures, and the intricate nature of protein–protein interactions.

Given the uncertainty of how a paratope engages and how multiple autoantigens can interact with the same autoantigen, one way to reduce the testing burden is to start with simpler multiepitope mRNA testing. 

Epitopes are the specific regions of antigens (in this case, the autoantibodies) that are recognized by autoantigens. For MHC binding, epitopes must bind to major histocompatibility complex (MHC) molecules to be presented to T cells. For class I MHC, epitopes are typically 8–11 amino acids in length, while for class II MHC, they are usually longer, around 15–24 amino acids. Some epitopes may be discontinuous and composed of amino acids not adjacent to the protein sequence. B cell epitopes are usually 5–17 amino acids in length but can be extended without adverse effects on the immune function [71,72].

## 4. Disease Targets

The mode of many ADs is not well established since often multiple ADs are involved, such as in the case of T1D patients exhibiting a presence of disease-associated serum autoantibodies [73]. T1D diagnosis is partially based on the detection of islet cell autoantibodies. The most often detected are insulin autoantibodies (IAA), anti-glutamic acid decarboxylase (GAD) autoantibodies, protein tyrosine phosphatase-related islet antigen 2 (IA-2) autoantibodies (also known as ICA512 autoantibodies) [74], and zinc transporter 8 (ZnT8) auto-antibodies [75]. However, some ADs have been well understood, and the autoantibodies that could be ideal targets for mRNA technology are listed in Table 1. This list includes only those ADs where the autoimmune etiology is well defined, and its associated autoantibodies recognized. These ADs represent the best target to create new mRNA therapies. 

An mRNA vaccine for each of these diseases can be readily designed by securing the sequence of the autoantibodies that are now treated as an “antigen” and idiotypic antibodies created by either determining the structure of the binding paratopes or by securing the epitopes of the autoantibodies and combining them in a single vaccine to provide broader protection against these ADs.

As an example, Q95LB0 APOH_PANTR (protein: Beta-2-glycoprotein 1; gene: APOH) binds to various kinds of negatively charged substances such as heparin, phospholipids, and dextran sulfate in the antiphospholipid syndrome. It prevents activation of the intrinsic blood coagulation cascade by binding to phospholipids on the surface of damaged cells. Its UniProt sequence is used to identify epitopes using IEBD [http://tools.iedb.org/main/ (accessed on 1 November 2023)] (Table 2).

Most vaccines induce antibody responses. Antibodies are the primary immune mechanisms that block infections. As we began to study nucleic acid vaccines, we discovered that because these vaccines are expressed within our cells, they are also very effective at inducing a T cell response. This discovery really prompted additional thinking about how researchers could use nucleic acid vaccines not just for infectious diseases, but also for immunotherapy to treat cancers and chronic infectious diseases—like HIV, hepatitis B, and herpes—as well as autoimmune disorders, and even for gene therapy.

The way to eliminate an autoimmune disorder is to modulate your immune cells to prevent them from attacking your proteins; in contrast to vaccines, the goal of which is to stimulate the immune system to recognize something better, treatment for autoimmune diseases seeks to dampen the immune system so that it stops attacking something it should not. Recently, researchers created an mRNA vaccine encoding a myelin protein with slightly tweaked genetic instructions to prevent it from stimulating immune responses. Instead of activating normal T cells that increase immune responses, the vaccine caused the body to produce T regulatory cells that specifically suppressed only the T cells attacking myelin (Table 2).

One way to create a universal vaccine will be to combine multiple epitopes, forming the open reading frame, into a single mRNA molecule, as shown in Table 3. The selection of multiple protein epitopes offers a creative opportunity, but it will require detailed studies of off-target events since many proteins acting as ADs may have multiple functions, some essential. However, in most instances, a careful analysis and identification of ADs specific to an autoantigen can be made.

**Figure 3:**
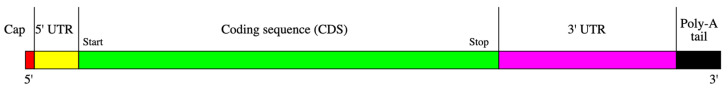
mRNA Structure. The 5’UTR (5’ untranslated region) cap refers to a modification at the beginning of a messenger RNA (mRNA) molecule. This modification, known as the 5’ cap, plays a crucial role in gene expression and translation. It is added to the 5’ end of the mRNA molecule during transcription to protect the mRNA from degradation, protect the mRNA from exonuclease, and facilitate the initiation of translation. It consists of a modified nucleotide called 7-methylguanosine (m7G) attached to the 5’ end of the mRNA through a unique 5’-5’ triphosphate linkage. This structure is often referred to as “m7G cap”. The cap is essential for the initiation of protein synthesis (translation). It serves as a recognition site for the ribosome, the cellular machinery responsible for translating mRNA into proteins. Some regulatory proteins can interact with the cap structure to modulate gene expression. Here is a sequence of 5′UTR cap: GAGAATAAACTAGTATTCTTCTGGTCCCCACAGACTCAGAGAGAACCCGCCACCATGTTCGTGTTCCTGGTGCTGCTGCCTCTGGTGTCCA. The Kozak sequence guides the pre-initiation complex (PIC) and ribosome to the translation initiation point (start codon) and facilitates ribosome assembly to ensure accurate translation of the protein sequence. The widely accepted Kozak sequence consensus is GCCGCCACCATGG, with ATG as the start codon. The Open Reading Frame serves as the translation code. The 3′-UTR directly succeeds the translation termination codon. The genetic material contains sections with regulatory functions, which affect gene expression after transcription. These regions also have binding sites for regulatory proteins and microRNAs (miRNAs). Through binding to the specific areas within the 3′-UTR, miRNAs can reduce the production of different mRNAs by either blocking translation or directly leading to the destruction of the transcript. The 3′-UTR contains silencer sequences that interact with repressor proteins, thereby suppressing the production of the mRNA. Several 3’-UTRs also have AU-rich elements (AREs). Proteins attach to AREs to influence the stability or rate of degradation of transcripts in a specific area or to impact the commencement of translation. In addition, the 3’-UTR region of the mRNA transcript contains the AAUAAA sequence, which guides the attachment of many adenine residues, known as the poly(A) tail, to the mRNA’s end. Poly(A) binding protein (PABP) attaches to this tail, playing a role in controlling mRNA translation, stability, and export. For instance, when the poly(A) tail of the mRNA binds to the poly(A) binding protein (PABP), it interacts with proteins that are linked to the 5’ end of the transcript. This interaction leads to the formation of a circular structure in the mRNA, which enhances the translation process.

The design of the mRNA molecule is shown in Figure 3.

### mRNA Delivery

To achieve the intended therapeutic benefits of mRNA, it is crucial for the mRNA molecule to successfully reach the target cell and generate enough of the required protein. The techniques and transportation of mRNA within cells are a substantial obstacle to the extensive implementation of mRNA therapies. The increased molecular size of mRNA (300–1500 kDa) poses a greater challenge for intracellular delivery compared to smaller molecules such as small interference RNA (siRNA) and anti-sense oligonucleotide (ASO) (4–14 kDa) [125]. Extracellular barriers comprise the cell membrane, made of a lipid bilayer of a zwitterionic negatively charged phospholipid. Additionally, ion pumps and channels uphold a negative potential, impeding mRNA entry into the cell. Exonucleases and RNases are found in the skin, blood, and extracellular barriers. These exonucleases degrade mRNA before its cellular uptake. 

An additional obstacle to the delivery of mRNA to the target cell is the presence of the cell membrane, which acts as a barrier and hinders the entry of mRNA into the cytoplasm. The cell membrane consists of phospholipids with negative charges and a lipid bilayer with positive and negative charges [126]. The mRNA molecules with a negative charge undergo repulsion from the negatively charged cell membrane. mRNA is vulnerable to destruction by ribonucleases in the extracellular milieu. Hence, the mRNA molecule needs to protect its negative charge from destruction by nucleases [127].

Various mRNA delivery techniques have been created, such as the direct administration of mRNA without any protective covering, lipid-based carriers, polymers, and protein derivatives [128,129]. 

## 5. Regulatory

As mRNA technology becomes more prevalent, regulatory agencies are developing guidelines and frameworks for evaluating and approving mRNA-based therapies; however, this remains unclear. Ensuring safety and monitoring any potential adverse effects are critical aspects of these regulatory processes [130].

The ICH Q3A and B guidelines mention “impurities in new drug substances and products produced by chemical synthesis.” However, it is essential to note that these guidelines do not apply to chemically synthesized oligonucleotides, including ASOs and siRNAs. Furthermore, it is worth noting that they do not adhere to the ICH Q6A guideline on “specifications: test procedures and acceptance criteria for new drug substances and new drug products: chemical substance.” The European Medicines Agency (EMA) classifies mRNA products as either gene therapy medicinal products (GTMPs) or vaccines based on their functions. In contrast, antisense oligonucleotides and RNA interference (RNAi) are categorized as chemicals according to the EMA’s classification system [131]. mRNA vaccines do not fall under the classification of “gene therapy” as they are specifically defined as “a medical intervention involving the alteration of genetic material within living cells [132]“. It is anticipated that the increasing number of product submissions will lead to the development of regulatory standards tailored specifically for therapeutic RNA products. 

A fundamental inquiry involving classifying mRNA products as chemicals or biologicals is necessary because many chemical drugs have biological functions. However, if the mRNA produces a protein, it should be qualified, or its translation outcome should be used to classify the mRNA.

## 6. Conclusions

The 100+ ADs present one of the direst needs for humanity to alleviate the suffering and the enormous cost burden. Though many temporary treatments for the alleviation of symptoms are available, a definitive treatment remains to be discovered. One such solution can be using anti-idiotypic antibodies that can mute the autoantibodies. However, given the diversified nature of autoantibodies involving multiple targets and multiple autoantibodies attacking the same autoantigens, and significant inter-patient variability, a more logical solution is to create universal vaccines that are based on the epitopes of the autoantibodies that can be either delivered in a single mRNA, or multiple molecules admixed in the formulation. Reaching out to the brain is also a better option using the mRNA formulations that are more likely to cross the BBB and reside primarily in the liver.

The flexibility of the design, the speed of development, the lower cost of growth, and the chemical nature that reduce the capital costs, besides the ability to deliver universal vaccines, make this an ideal solution to treat ADs. In conclusion, mRNA technology has several advantages compared to recombinant technology or in vitro translation techniques. 

It is anticipated that many new ventures will engage in this technology, given an almost unlimited potential that matches nicely with the humanitarian crisis caused by ADs and NDs that remain untreatable. It is indeed a paradigm shift of the best kind.

## Figures and Tables

**Table 1:** Diseases with confirmed autoimmune etiology and discovered autoantibodies.

Disease	Autoantibody
Acute motor axonal neuropathy [76]	ANCA, Anti-mitochondrial antibodies
Addison’s disease [77]	21-hydroxylase antibodies
Anti-NMDA receptor encephalitis [78]	Anti-GM1
Antiphospholipid syndrome [79]	Antiphospholipid antibodies
Autoimmune Encephalitis [80]	Anti-NMDA receptor
Autoimmune Pancreatitis [81]	IgG4, Anti-CA2 antibodies
Bickerstaff’s encephalitis [82]	Various, depending on subtype (e.g., NMDA receptor antibodies, LGI1 antibodies)
Bullous pemphigoid [83]	Anti-BP180, Anti-BP230
Celiac disease [84]	ANA, ASMA, anti-LKM1
Chronic inflammatory demyelinating polyneuropathy [85]	Anti-GQ1b
Cicatricial pemphigoid [86]	Anti-BP180, Anti-BP230
Dermatitis herpetiformis [87]	Anti-tissue transglutaminase
Dermatomyositis [88]	Anti-Jo1, Anti-Mi2, Anti-SRP, Anti-TIF1
Diabetes mellitus type 1 [89]	Anti-insulin, anti-IA-2, anti-GAD, anti-ZnT8 antibodies
Discoid lupus erythematosus [90]	ANA, Anti-dsDNA, Anti-Sm
Eosinophilic granulomatosis with polyangiitis (EGPA) [91]	Anti-streptolysin O (ASO), anti-DNase B
Epidermolysis bullosa acquisita [92]	Anti-type VII collagen
Gestational pemphigoid [93]	Anti-BP180, Anti-BP230
Goodpasture syndrome [94]	p-ANCA/MPO-ANCA
Granulomatosis with polyangiitis (GPA) [91]	ANCA
Graves’ disease [95]	TSI, TPO, TG antibodies
Graves’ ophthalmopathy [96]	TSH receptor antibodies
Guillain-Barré syndrome [97]	Various, including anti-MAG
Hashimoto’s thyroiditis [98]	TPO, TG antibodies
Hemolytic anemia [99]	Anti-red blood cell antibodies
IgA nephropathy [100]	Anti-GBM antibodies
Immune thrombocytopenia [101]	Anti-platelet antibodies
Lambert–Eaton myasthenic syndrome [102]	Various, including anti-GM1, anti-GD1a
Linear IgA disease [103]	Anti-epidermal basement membrane IgA
Lupus nephritis [104]	Anti-PLA2R antibodies
Lupus vasculitis	c-ANCA/PR3-ANCA
Lupus vasculitis [105]	ANA, anti-dsDNA, anti-Smith, others
Membranous nephropathy [106]	IgA autoantibodies
Microscopic polyangiitis (MPA) [107]	ANA, anti-dsDNA, anti-Smith, others
Myasthenia gravis	Anti-VGCC
Myasthenia gravis [108]	Anti-acetylcholine receptor, Anti-MuSK
Neuromyelitis optica [109]	Anti-AQP4, Anti-AChR, anti-MuSK; AQP4-IgG (NMO-IgG)
Ord’s thyroiditis [110]	TPO, TG antibodies
Paraneoplastic cerebellar degeneration [111]	Anti-Yo, Anti-Hu, Anti-Ri, others
Pemphigus vulgaris [112]	Anti-desmoglein 3, Anti-desmoglein 1
Pernicious anemia [113]	Anti-tissue transglutaminase antibodies (tTG), Endomysial antibodies (EMA), Deamidated gliadin peptide (DGP)
Polymyositis [114]	Anti-Jo-1, Anti-SRP, others
Premature ovarian failure [115]	Anti-ovarian antibodies, Anti-adrenal antibodies
Primary sclerosing cholangitis [116]	Anti-dsDNA, Anti-Sm, Anti-nuclear antibodies
Pulmonary alveolar proteinosis [117]	Anti-GM-CSF antibodies
Rheumatic heart disease [118]	Anti-IF, Anti-parietal cell
Rheumatoid lung disease [119]	Rheumatoid factor, Anti-CCP antibodies
Scleroderma (systemic sclerosis) [120]	ANA, anti-Scl-70, anti-centromere
Sjögren syndrome [121]	Anti-SSA/Ro, Anti-SSB/La antibodies
Stiff-person syndrome [122]	Anti-GAD, anti-amphiphysin
Sydenham’s chorea [123]	Anti-basal ganglia
Thrombotic thrombocytopenic purpura [124]	ADAMTS13 autoantibodies

**Table 2:** Epitopes of antiphospholipid syndrome autoantibody.

No.	Start	End	Peptide	Length
1	21	31	RTCPKPDDLPF	11
2	40	58	FYEPGEEITYSCKPGYVSR	19
5	119	135	ADSAKCTEEGKWSPELP	17
6	139	149	PITCPPPSIPT	11
7	157	166	KPSAGNNSLY	10
10	191	197	HGNWTKL	7
11	205	225	CPFPSRPDNGFVNYPAKPTLY	21
13	237	247	GYSLDGPEEIE	11
18	304	315	EKKCSYTEDAQC	12

**Table 3:** Open reading frame for a multi-epitope mRNA to translate a universal vaccine against diabetes type 1. The dash marks are to show the linker GSGSGSGS connecting the epitopes.

RTCPKPDDLPF-**GSGSGSGS**-FYEPGEEITYSCKPGYVSR-**GSGSGSG**S-ADSAKCTEEGKWSPELP-**GSGSGSGS**-PITCPPPSIPT-**GSGSGSGS**-KPSAGNNSLY-**GSGSGSGS**-HGNWTKL-**GSGSGSGS**-CPFPSRPDNGFVNYPAKPTLY-**GSGSGSGS**-GYSLDGPEEIE-**GSGSGSGS**-EKKCSYTEDAQC

## Data Availability

Not applicable.
